# ALA6, a P_4_-type ATPase, Is Involved in Heat Stress Responses in *Arabidopsis thaliana*

**DOI:** 10.3389/fpls.2017.01732

**Published:** 2017-10-04

**Authors:** Yue Niu, Dong Qian, Baiyun Liu, Jianchao Ma, Dongshi Wan, Xinyu Wang, Wenliang He, Yun Xiang

**Affiliations:** MOE Key Laboratory of Cell Activities and Stress Adaptations, School of Life Sciences, Lanzhou University, Lanzhou, China

**Keywords:** *Arabidopsis* P_4_-type ATPase, Aminophospholipid ATPase6 (ALA6), *ALA6* T-DNA insertion mutant (*ala6*), lipid flippase, membrane, heat stress response

## Abstract

Maintaining lipid membrane integrity is an essential aspect of plant tolerance to high temperature. P_4_-type ATPases are responsible for flipping and stabilizing asymmetric phospholipids in membrane systems, though their functions in stress tolerance are not entirely clear. Aminophospholipid ATPase6 (ALA6) is a member of the P_4_-type ATPase family, which has 12 members in *Arabidopsis thaliana*. Here, we show that a loss-of-function mutant of ALA6 (*ala6*) exhibits clear sensitivity to heat stress, including both basal and acquired thermotolerance treatments. Overexpression of *ALA6* improves seedling resistance to heat stress, while mutated *ALA6* transgenic plants, in which the conserved functional site of the ALA family has a point mutation, are still susceptible to heat stress like *ala6* loss-of-function mutant. In addition, *ala6* displays higher ion-leakage during heat treatment, suggesting that the lipid flippase activity of ALA6 plays a vital role in heat stress responses. Transcriptome analysis reveals differences in gene expression between *ala6* and wild-type plants with or without heat stress. The differentially expressed genes are involved primarily in the physiological processes of stress response, cellular compartment maintenance, macromolecule stability and energy production. Our results suggest that ALA6 is crucial for the stability of membrane when plants suffer from high temperature stress.

## Introduction

The phospholipid bilayer of cell membranes is asymmetrical, as the abundance of various lipid species on one side differs from that on the other. Within the membrane leaflets, the positions of the phospholipids are not fixed. Phospholipids perform multiple intramolecular motions, including rotation and lateral diffusion, and can also flip-flop between the two leaflets (Pomorski and Menon, 2016). The ambient temperature has a direct effect on the rate and frequency of these movements, eventually altering membrane fluidity. Thus, maintenance of the thermodynamic balance of the membrane is vital for cell function, especially during abrupt temperature changes (Murata and Los, 1997; Vigh et al., 1998, 2007; Horváth et al., 2012).

Studies have revealed that lipid asymmetry via flip-flopping across membrane leaflets is involved in numerous functions of the membrane system, including formation and maintenance of cell shape; vesicle budding; membrane trafficking, impermeability, and rigidity; extra- and intracellular signaling; fertilization; apoptosis; and membrane-coupling protein regulation (Puts and Holthuis, 2009; Andersen et al., 2016). Generally, the chemical traits of phospholipids dictate that transbilayer exchange of polar lipids should occur slowly and rarely. In fact, lipid translocation across the two leaflets is greatly assisted by flippases/floppases and/or scramblases. The membrane proteins that carry out these lipid translocation processes are classified into two categories based on whether the lipid transportation across the bilayer is driven by ATP (Pomorski and Menon, 2006, 2016). P-type ATPases are found in all kingdoms of life and possess a conserved aspartic acid residue within the P-type motif DKTGT that mediates reversible phosphorylation and conformational changes during substrate transport (Pedersen and Carafoli, 1987). Based on substrate specificity and sequence characteristics, P-type ATPases are divided into five subclasses, P_1_- through P_5_-type ATPases (Palmgren and Axelsen, 1998). P_4_-ATPases are flippases, which utilize the energy from ATP hydrolysis to transport specific phospholipids from the exoplasmic to the cytoplasmic face of the membrane against their concentration gradient (Coleman et al., 2009, 2013; Zhou and Graham, 2009; Lopez-Marques et al., 2014). These proteins are unique in that they flip “giant” phospholipids across membranes and are found only in eukaryotic cells (Paulusma and Elferink, 2010; Van Der Mark et al., 2013; Andersen et al., 2016). The first five P_4_-ATPases (Neo1p, Drs2p, Dnf1p, Dnf2p, and Dnf3p) were found in yeast, and at least 14 such proteins (ATP8A1 to ATP11C) occur in mammals (Paulusma and Elferink, 2010; Sebastian et al., 2012; Van Der Mark et al., 2013; Andersen et al., 2016). Most P_4_-ATPases have unique lipid specificities and subcellular localization. For example, Drs2p/ATP8A1 carries out PS and PE translocation in the late secretory pathway, while ATP8B2 and ATP10A were found to mediate PC asymmetry specifically (Ding et al., 2000; Alder-Baerens et al., 2006; Paterson et al., 2006; Xu et al., 2009; Zhou and Graham, 2009). Flippases with a relatively wide range of phospholipid specificities also exist, such as Dnf1p and Dnf2p, which can transport PC, lysophospholipids and synthetic alkylphospholipids in plasma membranes (Pomorski et al., 2003; Riekhof and Voelker, 2006; Baldridge et al., 2013).

Unlike those in yeast and mammals, the P_4_-ATPase in the plant *Arabidopsis thaliana* was identified much later and its function remains unclear. There are12 P_4_-ATPase proteins, ALA1 through ALA12 (Aminophospholipid ATPase subfamily) in *Arabidopsis* (Axelsen and Palmgren, 1998). Recent reports indicate that ALA2 internalizes PS in the endosomal system, whereas ALA3, localized in the Golgi apparatus, carries out flipping of a broad range of lipids, including PS, PE and PC (Poulsen et al., 2008; López-Marqués et al., 2010). ALA10 is located in the plasma membrane and internalizes various phospholipids, including lysoPC (Poulsen et al., 2015). With respect to biological function, ALA1 may play an important role in chilling tolerance (Gomès et al., 2000), while ALA2 may function with ALA1 in antiviral defense (Guo et al., 2017). ALA3 is involved in secretory processes of the Golgi apparatus at the root tip to regulate root growth (Poulsen et al., 2008). ALA6 and ALA7 are crucial for pollen fitness (McDowell et al., 2015). In addition, ALA10 has been found to function in leaf and root development, as well as in stomatal control (Poulsen et al., 2015; Botella et al., 2016). Some reports have also indicated that the physiological functions of several plant P_4_-ATPases can be affected by changes in temperature (Gomès et al., 2000; McDowell et al., 2013, 2015; Botella et al., 2016). Nevertheless, the ways in which these flippases respond to some types of temperature stresses remain unclear.

In the present study, our results suggest that seedlings of a loss-of-function mutant of *ALA6* (*ala6*) grow normally at standard temperatures, while they wither, turn yellow and eventually die under basal and acquired thermotolerance treatments. Overexpression of *ALA6* can improve plant tolerance of high temperature, and *ALA6* point-mutated transgenic plants lacking a conserved aspartic acid residue are still sensitive to heat stress. In addition, the results of a transcriptome analysis and observation of increased ion-leakage and *Chl b*/*a* ratios in *ala6* plants during heat treatment further suggest that ALA6 may protect plant cells from heat stress via the maintenance of membrane stability and integrity.

## Materials and Methods

### T-DNA Insertion Mutants

The *Arabidopsis thaliana* plants used here were in the Col-0 background. The T-DNA insertion mutant used in this experiment was *ala6* (SALK_150173), obtained from the Arabidopsis Biological Resource Center (Ohio State University^1^) with homozygous progeny determined via PCR screening (Alonso et al., 2003). The T-DNA insertion site is shown in **Figure 2A**, and all PCR primer sequences are given in Supplementary Table S1.

### Plant Growth Conditions

Seeds were surface-sterilized in 20% bleach for 12–15 min, rinsed five times with sterile water, and sown on ½ MS medium containing ½ Murashige and Skoog salts (MS; PhytoTech, Lenexa, KS, United States), 1% (w/v) sucrose, and 0.8% (w/v) agar. Plates were incubated at 4°C for 3 days in the dark and then transferred to a growth chamber at 21 ± 2°C with long days (16-h light/8-h dark cycles). The growth chamber was illuminated with white light at ∼110 μmol m^-2^ s^-1^.

### Gene Cloning and Transgenic Line Creation

Full-length cDNAs of *ALA6* and *ALA6* with a point mutation in the N-terminal aspartic acid at position 426 were amplified via PCR. These cDNA fragments were cloned into the pBIB binary vectors driven by the *ALA6* gene promoter. Four-week-old wild-type (WT) Col-0 and *ala6* mutant plants were transformed with *A. tumefaciens* (strain GV3101) using the floral dip method as described by Clough and Bent (1998). Homozygous transgenic plants were isolated on Basta. Four transgenic *Arabidopsis* lines were generated: complementary and overexpressing lines containing ALA6-autologous-promoter-ALA6, a tissue localization line containing the GUS gene driven by the ALA6-autologous-promoter, and a line expressing a point mutation in ALA6 driven by *ALA6* gene promoter. The point mutation was an A-to-C conversion at base 1277 relative to the start codon of the full-length *ALA6* cDNA (Supplementary Figure S1). All PCR primer sequences and vectors are shown in Supplementary Table S1.

### Basal and Acquired Thermotolerance Treatments

Thermotolerance assays were performed on 14-day-old seedlings as previously described (Larkindale and Vierling, 2008; Mittler et al., 2012). The basal heat treatment consisted of incubation at 43.5°C for 45, 60, or 90 min. The acquired thermotolerance treatment consisted of incubation at 37°C for 1.5 h followed by recovery at 22°C for 2 h and then by heat shock at 43.5°C for 1, 1.5, or 2 h. Plants were then moved to a growth chamber for 30, 36, or 72 h prior to sample preparation, measurement or photography. All assays were repeated at least in triplicate.

### Determination of Electrolyte Leakage

Fourteen-day-old seedlings were subjected to thermotolerance treatments and allowed to recover for 36 h. EL was determined as described by Sairam and Srivastava (2002). The sample (0.5 g) was placed in a 15-ml tube with 10 ml deionized water and incubated at 25°C for 2 h, and the conductivity in the solution was measured using a conductometer (R_1_). Samples were then heated for 15 min in a boiling water bath, and conductivity was measured again after the samples cooled to 25°C (R_2_). EL was calculated as the ratio of the initial conductivity to the conductivity after heating in boiling water (EL (%) = (R_1_/R_2_) × 100%). All assays were repeated at least in triplicate.

### Measurement of Chlorophyll Contents

Samples were collected from 50 to 100 mg leaves for chlorophyll extraction. Chlorophyll contents were measured using 95% ethanol as described previously (Woo et al., 2001). Leaf samples of 50–100 mg were placed in a test tube and pulverized in liquid nitrogen. The freeze-dried powder was incubated with 1 ml 95% ethanol in the dark at room temperature for 2–3 h. After dilution with solvent to 1.5 ml, the mixture was centrifuged at 13400 × *g* for 5 min. One milliliter of the supernatant was diluted 2- to 10-fold with 95% ethanol (OD value = 0.1–0.6), and the chlorophyll level was determined by a spectrophotometer at 665 and 649 nm. All steps above were performed at room temperature (25°C). The assay was repeated at least in triplicate. Chlorophyll contents were calculated using the following formulae: Chl a = 13.7 × OD_665_ - 5.76 × OD_649_, Chl b = 25.8 × OD_665_ - 7.6 × OD_649_, and Chl a + b = 6.10 ×OD_665_ + 20.04 ×OD_649_. *Chl a* is the chlorophyll *a* content, *Chl b* is the chlorophyll *b* content, *Chl a*+*b* is the total chlorophyll content, and OD is the absorbance.

### Semi-Quantitative RT-PCR

Fourteen-day-old WT and *ala6* seedlings grown on MS agar plates were collected, placed in tubes, and stored in liquid nitrogen. Total RNA was isolated using the MiniBEST Plant RNA Extraction Kit (Takara Biotechnology Co., Ltd., China). First-strand cDNA synthesis reaction was performed as Takara manual using Reverse Transcriptase M-MLV (RNase H-) (Takara Biotechnology Co., Ltd., China). PCR was carried out via a TP350 thermal cycler (Takara Bio Inc., Otsu, Japan) with the following program: 3 min at 94°C (1 cycle); 30 s at 94°C, 30 s at 53∼60°C and 30 s∼1.5 min at 72°C (28/32 cycles); and 5 min at 72°C (1 cycle). Each PCR reaction was replicated for three times. ACTIN2 was selected as a reference gene (see Supplementary Table S1 for primer sequences).

### Quantitative PCR

Fourteen-day-old WT, *ala6* and COM seedlings grown on MS agar plates were treated at 43.5°C for 45 min and recovered for 30 h, then collected in liquid nitrogen. Total RNA was reverse-transcribed into cDNA by the methods above. qPCR was carried out using a Stratagene MX3005P Real-Time System (Agilent, United States) with SYBR Premix Ex Taq (Takara Biotechnology Co., Ltd., China) as manufacturer’s instructions. Program for the reaction was as follows: 95°C for 30 s, 40 cycles of 95°C for 5 s, 60°C for 30 s. Melt curves (0.5°C increments in a 60–95°C range) were performed for each gene to assess the sample for non-specific targets and primer dimers. *UBQ11* was used as an internal control to normalize the expression of the target gene. A list of the primers used in these experiments is found in Supplementary Table S1. The ΔΔCt method was used to analyze relative transcript abundance (Rieu and Powers, 2009). The results were based on three independent experiments.

### GUS Staining

To identify the tissue-specific localization of ALA6, independent plants from each GUS-transgenic line were selected and various tissues were stained (Jefferson et al., 1987). Samples were incubated in a solution of 2 mM 5-bromo-4-chloro-3-indolyl-β-D-glucuronide (X-gluc), 3mM K_3_(Fe(CN)_6_), 3M K_4_(Fe(CN)_6_), 0.2% Triton-X-100, and 50 mM KH_2_PO_4_/K_2_HPO_4_(pH 7.2) at 37°C in the dark for overnight. Samples were then rinsed in 95% ethanol to remove chlorophyll. The samples were observed and photographed via anatomic microscope (SMZ-168, Motic Inc.).

### Transcriptome Analysis

Fourteen-day-old WT and *ala6* seedlings grown on MS agar plates were incubated at 43.5°C for 45 min and allowed to recover for 30 h. Gene expression analysis was performed by the Novogene Corporation, Beijing, China. RNA libraries were sequenced on a HiSeq 2500 instrument. TopHat version 2.0.12^2^ was used for mapping against the *Arabidopsis* genome^3^. HTSeq v0.6.1 was used to determine the read numbers for each gene. Based on the length of each gene and the corresponding number of reads, the FPKM value of each gene was calculated. Three independent experiments were conducted, and only genes having consistent expression changes in the three microarray assays were reported. DEG analysis was performed using the DESeq R package (1.18.0) with an adjusted *P*-value (<0.05). In addition, Gene Ontology (GO) enrichment analysis of DEGs was carried out using the GOseq R package, in which results were corrected for gene length bias. GO terms with corrected *P*-values less than 0.05 were considered to be significantly enriched DEGs.

### Statistical Analysis

Each experiment was repeated at least three times. Data were analyzed using one-way ANOVA with Turkey’s multiple-comparison test under a 0.05 confidence coefficient.

## Results

### Tissue Expression Pattern of *ALA6*

**FIGURE 1:**
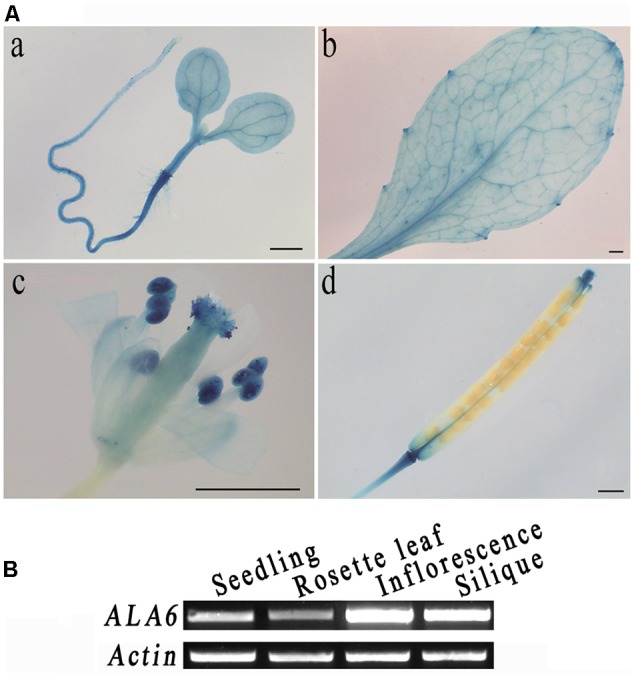
Tissue-specific expression of *ALA6*. **(A) a**, 5-day-old seedling. **b**, rosette leaf. **c**, flower, stamen, and pistil. **d**, silique. **(B)** ALA6 expression in various tissues via RT-PCR assay.

To analyze the tissue specificity of *ALA6*, its promoter region was fused to a *GUS* reporter gene and stably expressed in *Arabidopsis* plants. After staining, GUS signals were found throughout the young seedling except in the root tip, with particularly strong expression at the junction between the root and hypocotyl (**Figure 1A(a)**). In the leaf, the signal penetrated the veins and was especially strong in the leaf margin (**Figure 1A(b)**). In the flower and silique, staining was strong in the stigma, anther and base and tip of the silique; the signal was also particularly intense in pollen (**Figure 1A(c,d)**). Gene expressed in different tissues via semi RT-PCR analysis also showed that *ALA6* was expressed in reproductive organs more than that in vegetative tissues (**Figure 1B**). The expression of *ALA6* in numerous tissues suggests that ALA6 may be involved in multiple physiological activities in the plant.

### Loss of Function of ALA6 Confers Hypersensitivity to Heat Stress in *Arabidopsis* Seedlings

**FIGURE 2:**
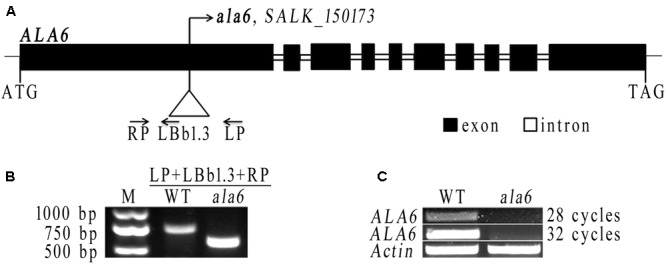
The T-DNA insertion line of *ALA6*. **(A)** Diagram of the *ALA6* gene and the T-DNA insertion site. **(B)** Identification of homozygous *ala6* mutants, using the primers shown in **(A)**. **(C)** Semi-quantitative analysis demonstrates that *ALA6* is knocked-out in the *ala6* mutant. 28 cycles and 32 cycles are cycles in PCR amplification.

To determine the function of ALA6, a T-DNA insertion mutant was obtained from the SALK collection (SALK_150173). The T-DNA was inserted into the first exon of *ALA6* (**Figure 2A**). The homozygous F2 progeny of *ala6* plants were screened (**Figure 2B**) and tested to determine *ALA6* expression levels, and it was found that the expression of *ALA6* in this T-DNA insertion line was completely suppressed (**Figure 2C**).

**FIGURE 3:**
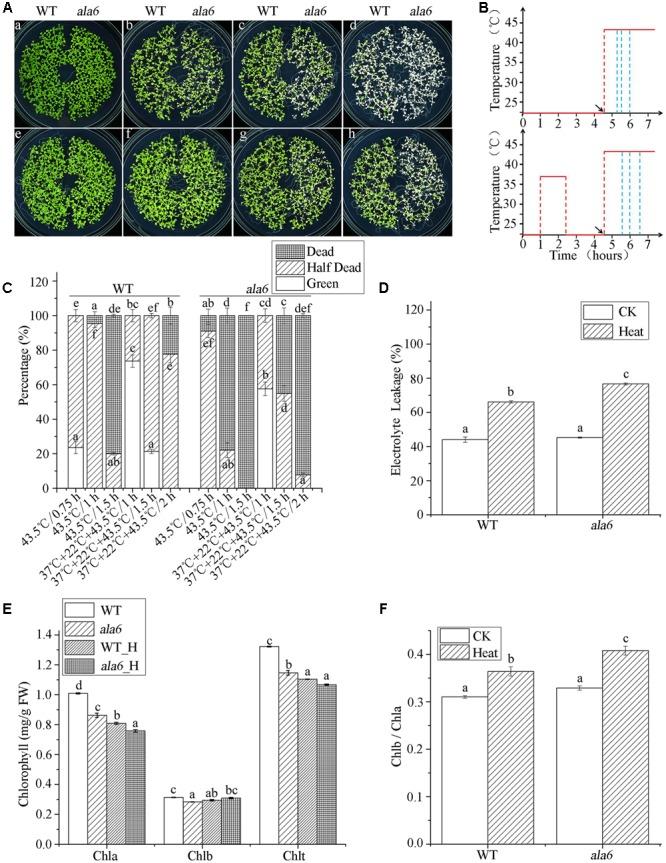
*ala6* is hypersensitive to heat stress. **(A) a** and **e** are 14-day-old WT and *ala6* seedlings without thermal treatment; **b** was incubated at 43.5°C for 45 min; **c** and **f** were incubated at 43.5°C for 1 h received prior heat acclimation at 37°C for 1.5 h; **d** and **g** received the same treatments as c and f, respectively, but the heat shock time was 1.5 h; **h** received acclimation at 37°C prior to heat treatment for 2 h. **(B)** Schematic diagram for heat treatment corresponding to **(A)**; from top to bottom, linear graphs represent basal thermotolerance (no acclimation) and acquired thermotolerance (acclimation). Red lines indicate the temperatures and blue lines indicate different treatment times. Arrows illustrate the times of the heat shock at 43.5°C. **(C)** The percentage of seedlings, including dead, half-dead and alive. The six columns correspond to b, c, d, f, g, and h in **(A)** are calculated from three independent experiments (average results ±SE). Columns with different letters have significantly different values (*P* < 0.05). **(D)** Electrolyte leakage (%) of 14-day-old seedlings treated at 43.5°C for 45 min and allowed to recover for 36 h. **(E)** Chlorophyll contents (mg/g) of seedlings as in **(D)**. WT_H, wild type treated with heat stress; *ala6*_H, *ala6* treated with heat stress; Chla, chlorophyll a; Chlb, chlorophyll b; Chlt, total chlorophyll. **(F)** Chlb/Chla ratio of **(E)**. Results are calculated from three independent experiments (average results ±SE). Columns with different letters have significantly different values (*P* < 0.05).

As ALA6 has been predicted to function in response to high temperature stress, two different experiments were performed to determine the heat-sensitive phenotype of *ala6* at a lethal temperature of 43.5°C (**Figure 3B**). As shown in **Figure 3A**, the first treatment consisted of incubation at high temperature for different lengths of time (45, 60, or 90 min) followed by recovery for 3 days. Compared to WT, *ala6* was extremely sensitive to heat stress. In the 45-min treatment, some *ala6* seedlings began to wilt, with leaves shrinking, curling, and yellowing. Seedlings also grew more slowly or died (**Figure 3A(b)**). After longer heat-treatments, the lethal effect of the *ala6* mutation was more obvious (**Figure 3A(c,d)**).

The second heat treatment consisted of exposure to a constant, non-lethal temperature (37°C) to allow acclimation, followed by incubation at an optimum temperature (22°C) prior to exposure to a lethal temperature (43.5°C) (**Figure 3B**). As shown in **Figure 3A(g,h)**, *ala6* remained sensitive to high temperature. Compared with the basal heat-treatment, both WT and *ala6* plants showed improved tolerance to high temperature after a brief period of acclimation. With preliminary heat acclimation, most WT seedlings survived, and approximately 50% of *ala6* seedlings turned yellow after 90 min at 43.5°C (**Figures 3A(g),C**). Without previous heat acclimation, only a few WT seedlings showed any viability, and all *ala6* seedlings were dead after 90 min at 43.5°C (**Figures 3A(d),C**). These results indicated that plants exposed to non-lethal temperatures in advance of abrupt exposure to high temperature showed improved responses to heat stress. In addition, it appeared that *ala6* was inherently heat-sensitive, showing susceptibility to heat treatment even after warm acclimation. ALA6 might play a crucial role in plant heat-tolerance.

Considering the conserved function of the ALA family in phospholipid transport, the membrane status of *ala6* plants under hyperthermia was investigated. WT and mutant seedlings were grown on MS plates for 14 days and incubated at 43.5°C for 45 min, followed by a 36-h recovery under optimum conditions. After this treatment, the growth of all seedlings was affected. Seedlings were collected, and EL was quantified; the results were shown in **Figure 3D**. Heat treatment significantly increased the rate of EL in both WT and mutant plants. Leakage rates increase by 48.9 and 68.9% due to heat treatments in WT and *ala6* seedlings, respectively. Notably, the membrane status of *ala6* cells was altered more than that of WT cells, implying that ALA6 might protect plant cells from heat stress by regulating plasma membrane permeability and stability.

Changes in the contents of photosynthetic pigments (chlorophylls) and in *Chl b*/*a* ratios are useful indicators of stress and tolerance in plants (Zhang et al., 2008). We found that chlorophyll levels decreased during heat stress treatments in both WT and *ala6* mutant plants, indicating possible heat-induced damage to the photosynthetic activity of the chloroplasts (**Figure 3E**). In addition, the *chl b*/*a* ratio increased dramatically in *ala6* mutants after basal heat treatment, reaching a level 36.4% higher than in *ala6* mutants without treatment, whereas the ratio in WT increased only 28.2% (**Figure 3F**). These results indicated that a lack of ALA6 might increase the injurious impact of heat stress on membranous organelles.

### The Intrinsic Function of ALA6 Is Critical for Thermotolerance

**FIGURE 4:**
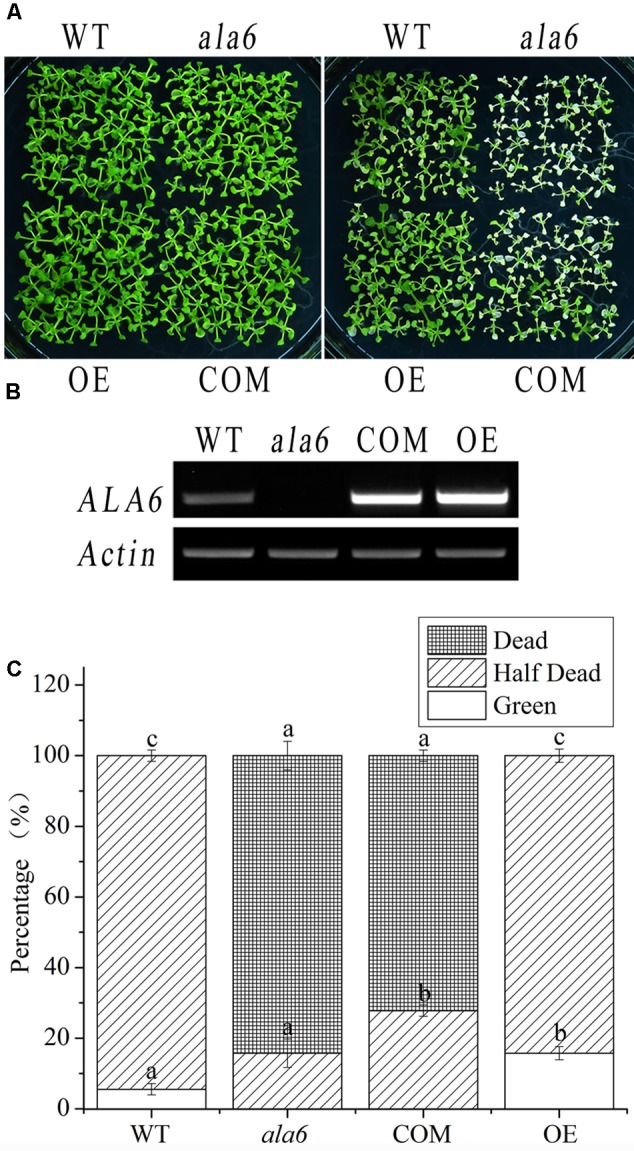
Complementation and overexpression lines of *ALA6* under basal thermotolerance. **(A)** Phenotypes of complementation and overexpression lines under heat stress. WT, wild type; *ala6, ALA6* T-DNA insertion line; COM, complementation line of *ALA6*; OE, overexpression line of *ALA6*. **(B)** RT-PCR analysis of *ALA6* expression in complementary and overexpressed transgenic lines. **(C)** Survival rate of seedlings corresponding to **(A)**. Results are calculated from three independent experiments (average results ±SE). Columns with different letters have significantly different values (*P* < 0.05).

To confirm the function of *ala6* under high temperature, an exogenous *ALA6* gene (normal or deficient in conserved gene function) was expressed in *ala6* and WT plants. The complementation lines resulting from the former transformation were subjected to heat treatment, as shown in **Figure 4**, and it was found that the expression of *ALA6* could rescue the heat-lethal phenotype of the loss-of-function mutant. The growth of the complementation lines resembled that of WT plants, even after heat treatment. In addition, the *ALA6* overexpression lines exhibited greater vitality than WT plants (**Figures 4A,C**), showing that ALA6 was involved in intracellular responses to heat stress, and the heat-sensitive phenotype of *ala6* was due to the loss of function of ALA6.

**FIGURE 5:**
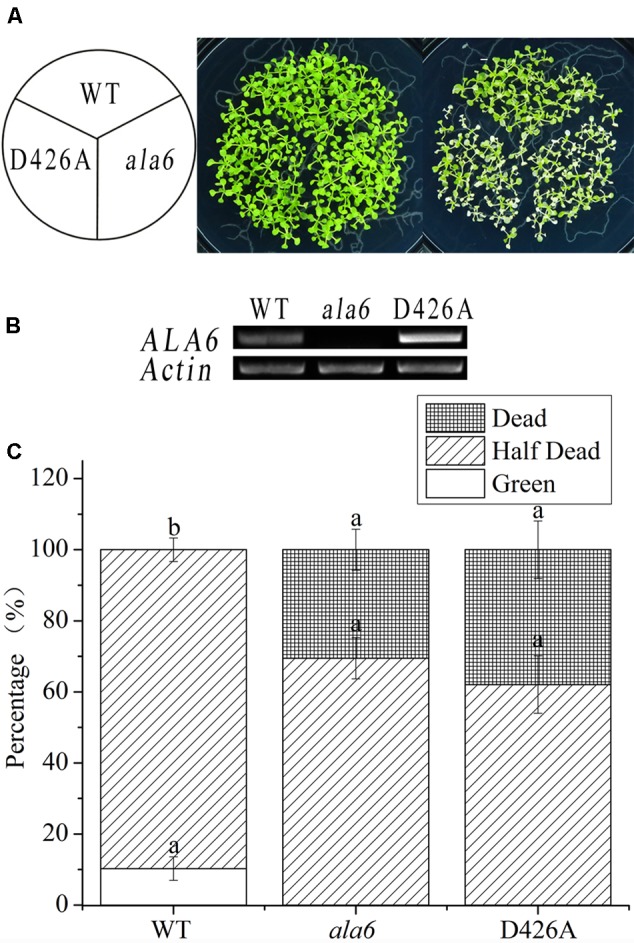
Point-mutant lines of *ALA6* under basal thermotolerance. **(A)** Phenotype of point-mutant lines under heat stress. WT, wild type; *ala6, ALA6* T-DNA insertion line; D426A, conserved aspartic acid-point mutant line of ALA6. **(B)** RT-PCR analysis of the expression of *ALA6* in point-mutant transgenic lines. **(C)** Survival rate of seedlings corresponding to **(A)**. Results are calculated from three independent experiments (average results ±SE). Columns with different letters contain significantly different values (*P* < 0.05).

P-type ATPases, including the ALA family in *Arabidopsis*, contain a motif that carries out phosphorylation during phospholipid transport; this motif contains an aspartic acid residue (Pedersen and Carafoli, 1987). To determine the relationship between the phospholipid transport activity of ALA6 and the heat stress response, transgenic lines containing a point mutation in the phosphorylation motif were generated. This mutation converted an A to a C in the codon encoding the conserved aspartic acid residue of ALA6. As expected, the transgenic lines showed a heat-hypersensitive phenotype like that of *ala6* under high-temperature treatment (**Figure 5A**). This result further demonstrated that the native phospholipid transport activity of ALA6 was implicated in heat-tolerance, and the specific phenotype of *ala6* was associated with a defect in its enzymatic activity. The levels of *ALA6* transcript in these transgenic lines were measured and were significantly higher than those of the WT (**Figures 4B, 5B**). This observation was also supported by the survival rate shown in **Figures 4C, 5C**.

### Microarray Analysis Reveals ALA6-Related Genes

**FIGURE 6:**
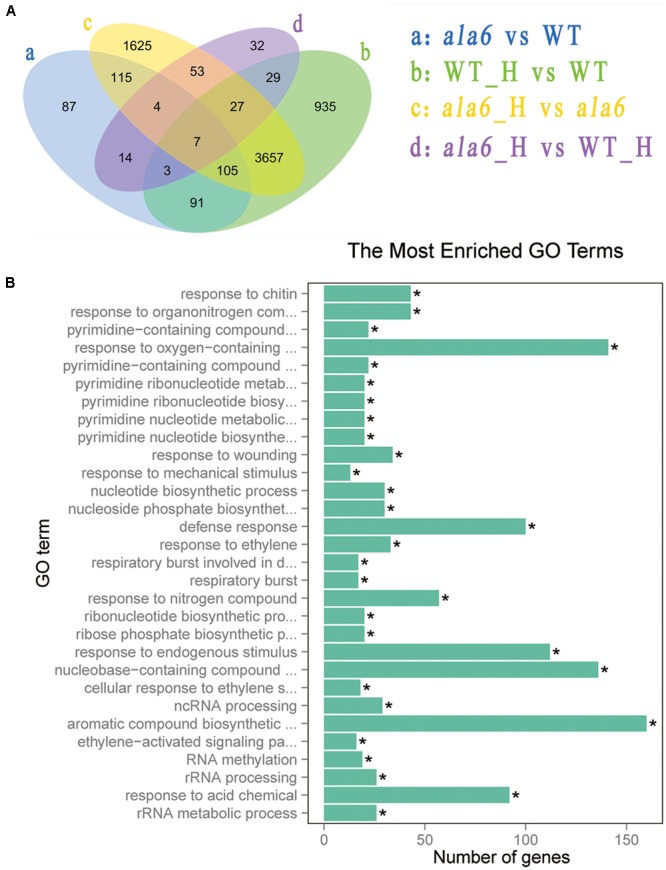
Differentially expressed genes after basal thermotolerance. **(A)** Venn diagram indicates DEGs. DEGs for a: *ala6* vs. WT; b: WT with heat treatment vs. WT; c: *ala6* with heat treatment vs. *ala6*; d: *ala6* with heat treatment vs. WT with heat treatment. **(B)** GO enrichment for DEGs.

To investigate the underlying mechanism of heat sensitivity in *ala6*, we analyzed the RNA-seq data of WT and *ala6 Arabidopsis* samples. A total of 426 DEGs were found to be related to ALA6 (**Figure 6A**). Of these, 286 genes showed down-regulated and 140 showed up-regulated expression in *ala6*. All the transcripts listed here exhibited twofold or larger changes in abundance. These DEGs were enriched in GO terms containing genes responsive to endogenous stimuli, chemical or organic substances, and biotic or abiotic stresses (shown in Supplementary Table S2). The top 30 DEGs that were annotated as stress response elements include various transcription factors, membrane components and transporters (**Table 1**). These results indicated that ALA6 might be involved in multiple cellular responses.

**Table 1:** Differentially expressed genes (DEGs) between WT and *ala6.*

Gene_id	FPKM_*ala6*	FPKM_WT	padj	Gene name	Description
AT1G54280	11.32	0.14	0	ALA6	ATPase E1-E2 type family protein/haloacid dehalogenase-like hydrolase family protein
AT1G25560	14.21	85.50	6.81*E*-275	TEM1	AP2/B3 transcription factor family protein
AT1G68840	17.46	90.52	1.48*E*-253	RAV2	Related to ABI3/VP1 2
AT5G21940	86.36	369.80	2.01*E*-233	–	Function unknown
AT2G40000	31.13	205.38	2.60*E*-224	HSPRO2	Ortholog of sugar beet HS1 PRO-1 2
AT1G23390	21.13	97.82	9.04*E*-222	–	Kelch repeat-containing F-box family protein
AT1G80440	36.24	160.52	2.97*E*-208	–	Galactose oxidase/kelch repeat superfamily protein
AT4G37610	17.23	90.47	1.18*E*-203	BT5	BTB and TAZ domain protein 5
AT1G73120	11.68	80.61	4.41*E*-175	–	
AT3G59940	62.83	225.29	1.64*E*-144	KFB50	Galactose oxidase/kelch repeat superfamily protein
AT5G60680	22.32	87.36	5.12*E*-140	–	Protein of unknown function, DUF584
AT3G46600	14.44	49.61	1.27*E*-138	–	GRAS family transcription factor
AT5G19120	78.50	218.55	1.62*E*-134	–	Eukaryotic aspartyl protease family protein
AT1G27730	11.42	59.58	1.85*E*-129	STZ	Salt tolerance zinc finger
AT4G05070	71.27	250.05	1.05*E*-128	–	Wound-responsive family protein
AT3G20340	12.07	63.50	1.56*E*-123	–	
AT1G68520	87.40	219.95	1.10*E*-122	BBX14	B-box type zinc finger protein with CCT domain
AT2G40140	24.02	82.13	5.15*E*-118	CZF1	Zinc finger (CCCH-type) family protein
AT5G37260	25.25	79.14	1.41*E*-107	CIR1	MYB family transcription factor Circadian 1
AT5G56550	11.66	80.83	1.35*E*-93	OXS3	Oxidative stress 3
AT5G61160	24.42	62.07	1.91*E*-83	AACT1	Anthocyanin 5-aromatic acyl transferase 1
AT5G24030	98.83	37.43	3.06*E*-83	SLAH3	SLAC1 homolog 3
AT1G80920	90.37	253.72	1.09*E*-81	J8	Chaperone DnaJ-domain superfamily protein
AT2G23130	114.40	37.32	9.69*E*-74	AGP17	Arabinogalactan protein 17
AT2G21210	13.92	47.65	4.39*E*-73	–	SAUR-like auxin-responsive protein family
AT1G32920	57.06	142.22	1.48*E*-69	–	
AT3G15450	149.28	423.60	1.58*E*-67	–	Aluminum induced protein with YGL and LRDR motifs
AT3G15630	86.45	183.59	2.66*E*-66	–	
AT5G67420	18.34	69.95	6.23*E*-66	LBD37	LOB domain-containing protein 37
AT5G18670	4.21	14.83	1.75*E*-65	BMY3	Beta-amylase 3

*Gene_id: Gene identity; FPKM: Fragments Per Kilobase of transcript per Million fragments mapped; padj: *P*-value by adjustment.*

**FIGURE 7:**
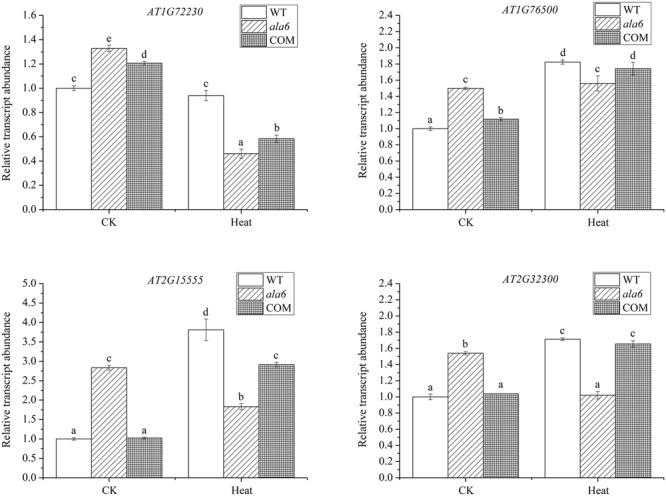
Relative transcript abundance of DEGs randomly selected in WT, *ala6*, and ALA6 complementary lines with or without heat stress. Results are calculated from three independent experiments (average results ±SE). Columns with different letters contain significantly different values (*P* < 0.05).

To identify genes related to ALA6 under heat stress, the RNA-seq data of *Arabidopsis* WT and *ala6* samples with and without heat treatment were analyzed as described in the Section “Materials and Methods.” Quantitative PCR analyzing the expression of several DEGs in WT, *ala6*, ALA6 complementary transgenic lines with or without heat stress also confirmed the results from RNA-seq data above (**Figure 7**). **Figure 6A** showed the DEGs for *ala6* vs. WT (a), WT with heat treatment vs. WT (b), *ala6* with heat treatment vs. *ala6* (c) and *ala6* with heat treatment vs. WT with heat treatment (d). It was believed that genes related to ALA6 under heat treatment should be found in b but not in c (**Figure 6A**). A total of 1058 DEGs were identified and implicated in heat stress responses related to ALA6. The top 30 DEGs in **Table 2** are involved in transcription factor activity, organelle components, and signal transduction and substrate transport. Of these genes, two transcription factors [*BBX14* (AT1G68520) and *ERF104* (AT5G61600)] had previously been implicated in stress response. And most of genes we screened were involved in photosynthesis-related activities (AT1G16720, AT4G12800, AT1G52870, AT2G30570, and AT1G06430) and transmembrane transport activity (AT5G49730, AT1G64720, AT5G46110, AT4G25570, and AT3G13062).

**Table 2:** Genes related to ALA6 under basal thermotolerance.

Gene_id	FPKM_*ala6*	FPKM_WT	padj	Gene name	Description
AT4G01560	0.39	31.34	4.01*E*-236	MEE49	Ribosomal RNA processing Brix domain protein
AT1G16720	151.56	65.46	1.23*E*-186	HCF173	High chlorophyll fluorescence phenotype 173
AT1G52930	0.44	28.23	1.03*E*-180	–	Ribosomal RNA processing Brix domain protein
AT5G21940	91.16	369.80	1.33*E*-179	–	
AT4G12800	2143.26	1009.12	4.42*E*-179	PSAL	Photosystem I subunit l
AT5G56010	66.77	236.09	5.39*E*-179	HSP81-3	Heat shock protein 81-3
AT5G49730	51.63	18.26	1.62*E*-175	ATFRO6	Ferric reduction oxidase 6
AT1G64720	790.28	326.70	8.54*E*-172	CP5	Polyketide cyclase/dehydrase and lipid transport superfamily protein
AT1G68520	44.88	219.95	4.48*E*-171	BBX14	B-box type zinc finger protein with CCT domain
AT5G46110	595.47	329.87	5.18*E*-143	APE2	Glucose-6-phosphate/phosphate translocator-related
AT3G15630	43.84	183.59	4.89*E*-138	–	
AT2G30600	82.35	40.48	3.19*E*-132	–	BTB/POZ domain-containing protein
AT1G21130	364.42	192.73	2.53*E*-124	–	O-methyltransferase family protein
AT1G52870	116.67	56.31	9.62*E*-120	–	Peroxisomal membrane 22 kDa (Mpv17/PMP22) family protein
AT5G19120	70.83	218.55	3.29*E*-118	–	Eukaryotic aspartyl protease family protein
AT3G13180	0.80	14.02	7.62*E*-117	–	NOL1/NOP2/sun family protein/anti-termination NusB domain-containing protein
AT2G30570	2336.70	1219.77	1.44*E*-112	PSBW	Photosystem II reaction center W
AT4G34710	101.81	275.88	6.62*E*-110	ADC2	Arginine decarboxylase 2
AT5G35170	137.68	76.90	4.15*E*-109	–	Adenylate kinase family protein
AT3G25770	60.87	197.45	4.99*E*-109	AOC2	Allene oxide cyclase 2
AT5G61600	10.47	62.01	1.14*E*-108	ERF104	Ethylene response factor 104
AT3G20340	7.86	63.50	1.02*E*-106	–	
AT4G25570	335.27	194.81	1.78*E*-103	ACYB-2	Cytochrome b561/ferric reductase transmembrane protein family
AT1G06430	86.33	46.32	1.79*E*-103	FTSH8	FTSH protease 8
AT4G37300	357.03	209.52	2.41*E*-103	MEE59	Maternal effect embryo arrest 59
AT3G13062	71.42	34.92	1.33*E*-101	–	Polyketide cyclase/dehydrase and lipid transport superfamily protein
AT5G58330	197.06	113.47	1.69*E*-99	–	Lactate/malate dehydrogenase family protein
AT1G63780	0.34	14.25	1.03*E*-98	IMP4	Ribosomal RNA processing Brix domain protein
AT4G37610	22.21	90.47	8.19*E*-98	BT5	BTB and TAZ domain protein 5
AT1G58280	38.96	12.63	1.42*E*-96	–	Phosphoglycerate mutase family protein

*Gene_id: Gene identity; FPKM: Fragments Per Kilobase of transcript per Million fragments mapped; padj: *P*-value by adjustment.*

Differentially expressed genes from the basal heat treatment were subjected to GO enrichment analysis (**Figure 6B**). The genes associated with ALA6 under heat stress included 112 genes categorized as “response to endogenous stimulus” and 194 categorized as “response to stress.” Other categories of enriched genes include “response to oxygen-containing compound” (141 genes), “defense response” (100 genes), “response to acid chemical” (92 genes), and “aromatic compound biosynthetic process” (160 genes). These results indicated that ALA6 might have versatile effects on cellular responses triggered by heat stress.

## Discussion

### ALA6 Is Involved in Heat-Induced Cellular Responses via Maintenance of Membrane Stability

Temperature is a major environmental cue that has a rapid impact on cellular homeostasis, including both membrane stability and protein activity, and also has significant effects on plant development and growth. Because land plants are sessile, they are exposed to daily and seasonal temperature fluctuations (Saidi et al., 2011). To survive, plants evolved a series of mechanisms to respond to the ambient environment, including membrane-related stress signaling mechanisms for intact cell (Vigh et al., 2005, 2007). Stress-triggered changes in the lipid species of membranes could influence both the physical properties of the membrane and the distribution and activity of membrane proteins (Vigh et al., 2005). P_4_-type ATPases have recently been found to alter membrane lipid composition by transporting specific lipids, and some members of this protein family have been found to play crucial roles in stress responses in yeast and mammals (Axelsen and Palmgren, 2001; Sebastian et al., 2012). Among the 12 members of the P_4_-type ATPase family in *Arabidopsis*, ALA1 was first reported to be implicated in cold tolerance (Gomès et al., 2000). In addition, pollen tube development in an *ala6/7* double mutant is described to be vulnerable under hot-day/cold-night temperature stress (McDowell et al., 2015). ALA3 has been demonstrated to participate in secretory processes of the Golgi apparatus, regulating root growth and reproductive development as well as tolerance to temperature stress (Poulsen et al., 2008; McDowell et al., 2013). Though ALA10 is responsible for root and leaf development independent of temperature, it can improve MGDG synthesis at low temperature (Poulsen et al., 2015; Botella et al., 2016). However, the functions of other members of this subclass have not been determined. In fact, the members of the ALA family that exhibit lipid substrate specificity have been shown to be involved in many physiological processes in a temperature-dependent manner. Considering the relatively close phylogenetic relationship among ALA family members 3–10 (Poulsen et al., 2015), we chose to investigate ALA6 and found that this protein is essential for responses to high temperature stress. As shown in **Figure 2A**, though a short-term acclimation can alleviate heat injury to seedlings, two heat treatments lead to the death of *ala6* seedlings, whereas WT seedlings survive. In addition, the partially sterile siliques of *ala6* plants under heat stress (data not shown) are comparable to the description given by McDowell et al. (2015). Since plants also suffer other stresses while acquiring thermotolerance via heat acclimation (Larkindale and Vierling, 2008; Mittler et al., 2012), this study shows that ALA6 can be implicated directly in heat stress-related intracellular responses rather than in other stresses. To determine whether the enzyme activity of phospholipid translocation is involved in the heat stress response, ALA6 with a point-mutation at a conserved functional site was analyzed and was confirmed not to rescue the heat-sensitive phenotype of an *ala6* knockout mutant (**Figure 5A**). Taken together with the greater EL and higher *Chl b*/*a* ratio of *ala6* mutants (Demidchik et al., 2014; Bi et al., 2016), this result implies that ALA6 might serve as a membrane stabilizer via altering membrane permeability, especially during heat stress.

### ALA6 Can Induce Changes in the Expression of Heat Stress-Related Genes

Under high temperature, changes in lipid composition affect membrane dynamics and initiate the corresponding signal transduction pathways (Vigh et al., 1998). It has been reported that membrane fluidity can modulate the transcription of heat-inducible genes (Carratù et al., 1996; Horváth et al., 1998). To identify the downstream signaling triggered by *ALA6*, we analyzed gene expression in WT and *ala6* plants with and without heat shock. DEGs between WT and *ala6 Arabidopsis* were shown to be involved in many functions. Among the 286 down-regulated genes are numerous genes related to stress and hormone responses, including heat, cold, salt, drought, oxidative, and pathogen stress, as well as ethylene, abscisic acid, JA and salicylic acid in various other processes. These responses are involved in the control of gene expression by transcription factors, such as the F-box protein family, the B-box zinc finger protein family, *WRKY*s and *ERF*s (**Table 1**). For instance, the expression of *BBX14* (AT1G68520), a B-box-type zinc finger protein, was significantly lower in *ala6* than in WT *Arabidopsis*. Although the function of BBX14 is still unknown, expression of its homolog, *BBX18*, has been confirmed to be induced by heat shock and to affect seed germination and seedling survival negatively by suppressing the expression of heat-responsive genes (Wang et al., 2013). Other members of this family are also involved in responses to various biotic or abiotic stresses (Gangappa and Botto, 2014), likely explaining why *ala6* plants are sensitive to heat stress. In addition, it was found that 140 genes with up-regulated expression in *ala6* plant are responsible for various biological processes such as phosphorylation, substrate transport and synthesis, and lipid metabolism, but most of these genes have an integral membrane component (**Table 1** and Supplementary Table S2). Of these genes, only two fell within the top 30 DEGs with increased expression; these were *SLAH3* (AT5G24030) and *AGP17* (AT2G23130). The products of these genes both localize to the plasma membrane. SLAH3 is an anion channels involved in mediating ion homeostasis, while AGP17 is a glycosylphosphatidylinositol (GPI)-anchored protein involved in extracellular signal perception and interaction with proteins in lipid rafts (Gaspar et al., 2004; Zhang et al., 2016). The results imply that lack of ALA6 might not only lead to the alteration of membrane permeability and integrity but also to corresponding changes in membrane proteins.

Interestingly, the expression of many chloroplast-related genes in the top 30 DEGs are shown to be up-regulated in *ala6*, whose functions depend greatly on membrane stability (**Table 2**; Boudière et al., 2014). A *HSP81-3* (*HSP90.3*, AT5G56010), which functions as a molecular chaperone, and *AOC*2 (AT3G25770), which has AOC activity, are among the most interesting genes we identified. The major functions of *HSPs* include preventing proteins from misfolding and disaggregation and protecting membranes that are exposed to high temperature (Banti et al., 2010). Proteins such as HSP20, HSP70, HSP90 and HSP101 have been shown to be components of the heat shock response and assist in thermotolerance by adjusting protein aggregation (Nieto-Sotelo et al., 2002; Cazalé et al., 2009; Fragkostefanakis et al., 2015; Yu et al., 2016). Thus, the decreased expression of *HSP* genes in an ALA6 loss-of-function mutant may also demonstrate that ALA6 plays an important role in heat-inducible signaling. Moreover, AOC2 catalyzes the biosynthesis of JA, for which a role in basal thermotolerance has been illustrated recently (Clarke et al., 2009; Stenzel et al., 2012). In conclusion, when plants are exposed to high temperature, it is speculated that ALA6 may have an effect on the membrane permeability and stability, and this alteration can induce changes in membrane proteins and eventually trigger heat-associated gene expression. However, the physiological functions and underlying mechanisms of these altered genes in *ala6* mutants require further study.

## Conclusion

ALA6, a P_4_-type ATPase of *Arabidopsis*, possesses the conserved structure and function of this family and serves as a phospholipid flippase to maintain membrane stability and fluidity under normal or adverse conditions. The seedlings of loss-of-function mutant *ala6* were found to wither, turn yellow and eventually die with exposure to basal thermotolerance or acquired thermotolerance treatments. Overexpression of *ALA6* can rescue this phenotype, while transgenic plants containing a point-mutation in a conserved region of *ALA6* show no response to heat stress. This suggests that ALA6 is significant for response to high temperature stress. Moreover, *ala6* seedlings exhibit higher ion-leakage and lower chlorophyll content after heat treatment, indicating that ALA6 can protect membranes from the disordered state that results from heat stress. This protective effect involves the maintenance of membrane permeability, stability and integrity. In addition, transcriptome analysis shows that *ALA6* affects the expression of heat-inducible genes. Taken together, these evidences show that ALA6 plays an essential role in cellular responses to high temperature. In addition, characterization of ALA6 activity and its roles should further illuminate the mechanisms of the involvement of P_4_-ATPase in stress responses.

## Accession Number

RNA sequencing data is available at the National Center for Biotechnology Information (NCBI) data repository (accession PRJNA390831).

## Author Contributions

YN and YX designed research; YN, DQ, BL, JM, DW, XW, and WH performed research; YN, DQ, BL, JM analyzed data; and YN, DQ, and YX wrote the paper.

## Conflict of Interest Statement

The authors declare that the research was conducted in the absence of any commercial or financial relationships that could be construed as a potential conflict of interest.

## Footnotes

1 http://abrc.osu.edu/

2 http://tophat.cbcb.umd.edu/

3 www.arabidopsis.org

## Abbreviations

AGP: Arabinogalactan protein
AOC: Allene oxide cyclase
BBX: B-box domain protein
Chl: Chlorophyll
DEG: Differentially expressed gene
EL: Electrolyte leakage
ERF: Ethylene response factor
FPKM: Fragments per kilobase of transcript per million fragments mapped
GUS: β-Glucuronidase
HSP: Heat shock protein
JA: Jasmonic acid
MGDG: Monogalactosyldiacylglycerol
PC: Phosphatidylcholine
PCR: Polymerase chain reaction
PE: Phosphatidylethanolamine
PS: Phosphatidylserine
PSII: Photosystem II
SLAH: Slow anion channel-associated homolog
UBQ: Ubiquitin
WRKY: Transcription factor with conserved amino acid sequence WRKYGQK
