# Supplementary material for: ALA6, a P4-type ATPase, Is Involved in Heat Stress Responses in Arabidopsis thaliana
Source: Front Plant Sci. 2017 Oct 4;8:1732. doi: 10.3389/fpls.2017.01732 (PMC5632816; doi:10.3389/fpls.2017.01732)
Supplement: Supplementary file 1 [file Table_1.PDF]

Primers for identification

T-DNA insertion	LP	TTGACCTGAAGATGAAAACCG
	LBb1.3	ATTTTGCCGATTTCGGAAC
	RP	ACAAATCCTTTGTATGCGTGG
ALA6 (for semi-RT-PCR)	F	TTGACCTGAAGATGAAAACCG
	R	ACAAATCCTTTGTATGCGTGG
ACTIN2(for semi-RT-PCR)	F	TGCCAATCTACGAGGGTT
	R	CCTGCCTCATCATACTCG
AT1G72230 (for qPCR)	F	CACTACTTCATCTGCGGTATCC
	R	CCGTCTTGTACGGGTGTATTT
AT2G15555 (for qPCR)	F	GTTGGCTCGGGTAGTAAGAAA
	R	CCCAACATAGGGAGACAAGAAA
AT2G32300 (for qPCR)	F	GGGACTTCTCTGCCGTTATTC
	R	CGGTAGTACTAGAAGGGAAGGT
AT1G76500 (for qPCR)	F	CCTTGAAGATGAAGGAGGAGAAG
	R	CTCCAGATGGTGGTGATGATG
UBQ11 (for qPCR)	F	CTCTGACACCATTGACAACG
	R	TTTACGAAGATCTGCATACCTCC

Primers for cloning

ALA6-autologous-promoter	F	GGGGACAAGTTTGTACAAAAAAGCAGGCTTCGCAATTCTAGAGATACTCCTTGTT
	R	GGGGACCACTTTGTACAAGAAAGCTGGGTCTTTTCTCTTCGCCGCAATCGTATG
Point-mutation at D426A	F	GATACTATCCTTTCTGCTAAAACAGGAACTTTG
	R	CAAAGTTCCTGTTTTAGCAGAAAGGATAGTATC
Full-length cDNAs of *ALA6*	F	GGGGACAAGTTTGTACAAAAAAGCAGGCTTCATGGCTCGACGTAGAATAAGATC
	R	GGGGACCACTTTGTACAAGAAAGCTGGGTCTTGTGAGTTTGAAGAAGGTGTAT

Vectors

Pbib-A6P-Basta-GWR

Strains

DH5α(*E.coli*)

GV3101(*A. tumefaciens*)
