# Supplementary material for: ALA6, a P4-type ATPase, Is Involved in Heat Stress Responses in Arabidopsis thaliana
Source: Front Plant Sci. 2017 Oct 4;8:1732. doi: 10.3389/fpls.2017.01732 (PMC5632816; doi:10.3389/fpls.2017.01732)
Supplement: Supplementary file 3 [file Presentation_1.pdf]

Supplementary Material

ALA6, a P_4-type ATPase, is involved in heat stress responses in *Arabidopsis thaliana*

Yue Niu[1*], Dong Qian, Baiyun Liu, et al.

*** Correspondence:** Yue Niu, and Yun Xiang: niuy@lzu.edu.cn; xiangy@lzu.edu.cn

Table **S1 Primers, vectors and strains (1 file).**

Table S2 GO enrichment list for DEGs between WT and *ala6* (1 file).

Fig. S1 Amino acid sequences of ALA family members (1 file).
