# Supplementary figures and images for: ALA6, a P4-type ATPase, Is Involved in Heat Stress Responses in Arabidopsis thaliana
Source: Front Plant Sci. 2017 Oct 4;8:1732. doi: 10.3389/fpls.2017.01732 (PMC5632816; doi:10.3389/fpls.2017.01732)

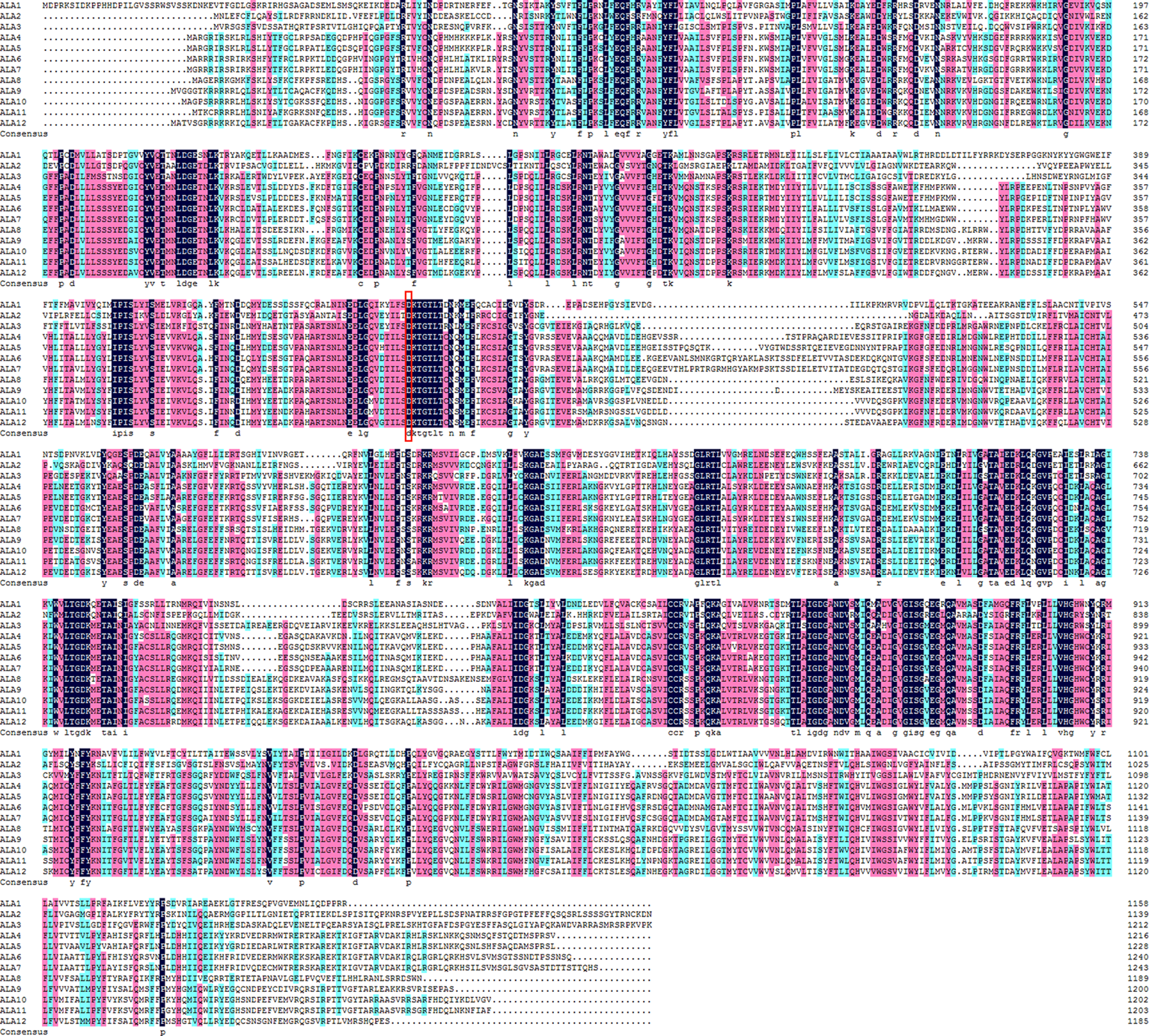

Supplement: Supplementary file 4 [file Image_1.TIF]
